# Insights into the interplay between stroke and depression through lipid metabolism-related diagnostic genes

**DOI:** 10.1186/s13041-026-01275-5

**Published:** 2026-01-18

**Authors:** Yun Liu, Bo Chen, Yu Yang, Yang Li, Xijuan Xia, Kehan Yan, Hu Xu, Yuefeng Li, Xin Tan

**Affiliations:** 1 Department of Radiology, Affiliated Yixing Hospital of Jiangsu University, Yixing, 214200 Jiangsu Province China; 2 Department of Neurosurgery, Affiliated People’s Hospital of Jiangsu University, Zhenjiang, 212001 Jiangsu Province China

**Keywords:** Stroke, Depression, Lipid metabolism, Machine learning, Neutrophil

## Abstract

Stroke, a result of acute cerebrovascular disease that causes cerebral dysfunction, often coexists with depression or even major depressive disorder (MDD). Despite the recognized significance of lipid metabolism disorders in both stroke and depression, their interwoven role in the pathogenesis of these conditions remains largely uncharted. This study sourced transcriptomic data linked to stroke and depression from the GEO database. Hub genes were identified through weighted gene coexpression network analysis (WGCNA) and machine learning algorithms. The diagnostic efficacy of the model featuring hub genes was evaluated using receiver operating characteristic (ROC) curve analyses and nomogram plots. Enrichment analysis and immune infiltration were examined while potential therapeutic agents were predicted using the drug profile database. The expression levels of the hub genes were verified on peripheral blood samples using quantitative Real-Time Polymerase Chain Reaction (qRT-PCR). 6 differentially expressed genes (DEGs) related to lipid metabolism were identified showing significant enrichment in metabolic and immune pathways. The diagnostic model constructed based on these genes demonstrated robust performance across multiple datasets. Gene set enrichment analysis (GSEA) suggested the involvement of nucleic acid metabolism and olfactory transduction in both diseases. Immune infiltration analysis revealed significant differences among various immune cells, such as monocytes and neutrophils. 11 potential drugs targeting at least two hub genes were identified. The exploration of lipid metabolism-related diagnostic genes offers valuable insights into the potential interplay between stroke and depression.

**Supplementary Information:**

The online version contains supplementary material available at 10.1186/s13041-026-01275-5.

## Introduction

The aging population is facing an escalating burden of neuropsychiatry diseases, imposing substantial economic strains on society. Stroke, a prevalent acute cerebrovascular condition, is associated with high rates of disability and mortality. Global statistics from 2016 indicate a staggering lifetime risk of stroke of approximately 25% for individuals aged 25 and above [1]. Depression is a common neuropsychiatric sequela after stroke, with prevalence ranging from 11% to 41% [2]. Post-stroke depression (PSD) is frequently encountered in clinical settings, with emerging evidence highlighting intricate connections between the onset and progression of both conditions. Notably, while stroke increases vulnerability to depression the reverse relationship holds true as well—depression precipitating cardiovascular events, strokes, and transient ischemic attacks [3]. PSD significantly worsens stroke prognosis by increasing morbidity and mortality rates [4]. Nonetheless, the precise mechanisms underlying the interplay between depression and stroke remain enigmatic.

Emerging research underscores the association between peripheral blood metabolic alterations and depression, particularly the tryptophan-kynurenine and fatty acid metabolism pathways [5]. Remarkably, up to 72.73% of MDD patients exhibit abnormal lipid metabolism, closely correlated with symptom severity [6]. Analogous changes in metabolite profiles are observed in the serum and plasma of stroke patients, with plasma metabolomics pinpointing amino acid metabolism, lipid metabolism, and oxidative stress as pivotal factors in PSD comorbidity [7, 8]. The convergence of metabolic disturbances, notably in lipid metabolism, between depression and stroke presents a promising avenue for further exploration.

Clinically, depression manifests through mood variations, psychomotor changes, cognitive impairments, and somatic or neurovegetative alterations. Approximately 30% of stroke survivors develop neuropsychiatric syndromes, with prevalent outcomes such as PSD, anxiety, fatigue, and apathy, mirroring overlapping symptoms of depression [9]. Both stroke and depression are multifaceted disorders influenced by diverse factors including genetics and environmental triggers. Heritability studies have demonstrated substantial genetic influences on depression and ischemic stroke, underscoring varying heritability rates across different stroke subtypes [10, 11]. In addition, dysregulated lipid metabolism in depression impacts enzyme activities, inflammatory responses, oxidative stress, and lipid peroxidation, thereby influencing disease progression [12]. Chronic exposure to substances such as acrylamide can exacerbate anxiety and depression symptoms by disrupting lipid metabolism and triggering inflammatory responses [13]. The intricate relationship between lipid metabolism, stroke, and depression reveals a compelling avenue for exploring the shared molecular pathways underlying these conditions.

Bioinformatics analyses have revealed the involvement of hypolipidemia in depression through the MANF/EWSR1/ANXA6 pathway [14] while microbiota-derived short-chain fatty acids are implicated in PSD through the modulation of lipid metabolism [15]. Apolipoprotein E (ApoE), a vital lipid carrier that promotes brain injury repair [16], has been linked to increased risks associated with both depression and stroke [17]. These insights underscore the profound influence of lipid metabolism on disease pathogenesis and treatment. Studies highlight the role of lipid metabolism in treatment efficacy such as KXS, demonstrating antidepressant effects through regulating signaling pathways related to lipid metabolism disorders [18]. Interleukin-33 (IL-33), mediated by lipogenesis, shows promise in enhancing blood‒brain barrier (BBB) repair and long-term functional recovery after stroke [19]. Specific lipids following brain injury may trigger autonomous neural repair processes [20]. Furthermore, differential gene expression related to lipid metabolism can predict responses to antidepressant therapies such as infliximab [21].

Given the key role of lipid metabolism in both stroke and depression, investigating genes common to this pathway across these diseases is crucial. By analyzing lipid metabolism-related genes (LMGs) in stroke and depression using bioinformatics tools and machine learning algorithms, researchers have aimed to identify potential diagnostic biomarkers and targeted therapeutics. This comprehensive approach offers new perspectives to advance our understanding of the shared molecular mechanisms underpinning stroke and depression.

## Methods

### Data collection and processing

We searched several gene expression datasets from the GEO database (https://www.ncbi.nlm.nih.gov/geo/), including GSE98793, GSE127711, GSE52790 of depression and GSE37587, GSE58294, GSE16561 of stroke. In addition, GSE32280, GSE39653, and GSE146882 were used for independent verification. The screening criteria for data of patients with depression are: (1) Moderate and severe depression; (2) No other mental disorders. The inclusion criteria for stroke patients are: (1) Patients with ischemic stroke; (2) Blood should be drawn within 24 h after the onset of symptoms. (3) No other neurological diseases. Detailed descriptive information of datasets was shown in Table 1. Datasets were merged and normalized to remove the batch effect between arrays by the “SVA” and “limma” packages in R. Differentially expressed genes were calculated between disease and control groups. The screening criteria were set as P value < 0.05 and |FoldChange|>1.1 in depression and stroke. The difference analysis results were presented in the form of the volcano plot and heatmap in which blue indicated low expression and red indicated high expression.

**Table 1:** Descriptive statistics of the GEO datasets

Diseases	GEO series	GPL platform	Sample information
Depression	GSE127711	GPL570	Depression: 112
	GSE98793	GPL570	Control: 64 VS Depression: 64
	GSE52790	GPL17976	Control: 12 VS Depression: 10
	GSE39653	GPL10558	Control: 24 VS Depression: 21
	GSE32280	GPL570	Control: 8 VS Depression: 8
Stroke	GSE58294	GPL570	Control: 23 VS Stroke: 23
	GSE16561	GPL6883	Control: 24 VS Stroke: 39
	GSE37587	GPL6883	Stroke: 34
	GSE146882	GPL23178	Control: 10 VS Stroke: 10

### Functional enrichment analysis

To explore the potential biological functions and signaling pathways of genes, we used the “clusterProfiler” R package to conduct Gene Ontology (GO) and Kyoto Encyclopedia of Genes and Genomes (KEGG) pathway enrichment analyses on the differentially expressed genes obtained from the analysis. GO analysis and KEGG enrichment analysis were statistically significant at *P* < 0.05 with false discovery rate (FDR) correction.

### Weighted gene co-expression network analysis

WGCNA is one of the most widely applied systems bioinformatics methods used to describe gene association patterns among different samples. It can cluster genes with similar expression patterns and analyze the association between modules and traits. The R package “WGCNA” is used to construct a co-expression network. Hierarchical clustering was performed to identify outliers. The appropriate soft threshold power (β = 1 in depression, β = 16 in stroke) is respectively set as the weight value in this study. The gene expression similarity matrix was converted into anadjacency matrix by “adjacency” function and converted again into a Topological Overlap Matrix (TOM). An hierarchical clustering tree was constructed and the dynamic tree cut algorithm (minModuleSize = 20 in depression and stroke) was used to find different modules. With the mergeCutHeight determined as 0.25 in both diseases, similar modules were merged in each group. Finally, screening the modules with the most significant positive and negative correlations of module-trait relationships according to pearson correlation.

### Lipid metabolism related genes

776 genes related to lipid metabolism were obtained from previous studies [22] and 22 lipid-metabolism-related pathways were extracted from Molecular Signature Database (MSigDB), including a total of 597 genes. By union, 875 genes related to lipid metabolism were obtained. After that, LMGs, DEGs, and key module genes from WGCNA in depression and stroke were intersected to identify the hub genes, by which we can further the study of the relationship between lipid metabolism and co-morbidity of depression and stroke.

### Machine learning algorithms

In this study, we performed three machine learning algorithms to screen and confirm the candidate hub genes for diagnosis, including the Least Absolute Shrinkage and Selection Operator (LASSO) logistic regression, Random Forest (RF) algorithm and Support Vector Machine-Recursive Feature Elimination(SVM-RFE). LASSO is a machine learning technique that combines variable selection and regularization, which can enhance predictive accuracy [23]. RF has the advantages of unconstrained variable conditions and superior accuracy, sensitivity, and specificity, so it is suitable for the prediction of continuous variables and can provide consistent forecasts [24]. The SVM-RFE algorithm is a widely applied supervised machine-learning protocol for classification and regression, which can identify genes with higher discriminative power [25]. The samples were randomly divided into training and test groups in a 5:5 ratio for both depression and stroke patients. We employed the R software’s “glmnet”, “randomforest”, and “e1071” packages to conduct LASSO, RF, and SVM analyses respectively. Cross-validation was carried out for all three algorithms. Finally, we got the intersection of the results of three algorithms above as the disease diagnostic target genes.

### Differential expression and diagnostic accuracy of hub genes

In the training and test groups, we analyzed the differential expression and diagnostic accuracy of hub genes. The “pROC”package was used to construct ROC curves to assess the accuracy of the diagnostic genes. In addition, we constructed the nomogram plot respectively to evaluate the diagnostic value of candidate genes in depression or stroke. 95% confidence interval and the area under the curve (AUC) which value > 0.7 can be considered to have a significant difference was generated through the ROC analysis. GSE32280, GSE39653 for depression and GSE146882 for stroke were used as external validation.

### Immune infiltration analysis

This study used an analytical tool named CIBERSORT to assess the abundance of immune cell subpopulations in samples by applying a deconvolution algorithm with gene expression data. We severally analyze the otherness of immune cells in peripheral blood between the diseased and healthy population in depression and stroke. At the same time, the correlations in immune cells were also analyzed. Furthermore, pearson correlation coefficient was used to determine the correlation between hub genes and immune cells.

### GSEA and prediction of the drug sensitivity

The study ranked the expression correlation of the target gene with all other genes in the entire genome, examined whether specific pathways were significantly enriched at the top or bottom of the ranking list, and screened out the upregulated and downregulated pathways that were significantly enriched in stroke and depression. The analysis results were corrected by FDR and sorted according to the normalized enrichment score (NES). The Drug Signature Database (DSigDB, https://maayanlab.cloud/Enrichr/) was used to explore potential drug molecules which can target diagnostic genes. As a publicly available web database obtained from the Enrichr web server, the DSigDB provides information for drugs and their target genes to analyze. Drug candidates were finally identified for the possible treatment of depression and stroke with the statistical threshold of p value < 0.05.

### Clinical sample validation of hub genes

Blood samples including 3 patients with depression, 3 stroke patients and 6 matched healthy controls were collected from Jiangsu University Affiliated Yixing Hospital to verify the data analysis results. This study has been approved by the Ethics Committee of Jiangsu University Affiliated Yixing Hospital. After centrifuging the whole blood sample, the serum was taken and stored at -80℃ until use. Total RNA was extracted using TRIzol reagent (Invitrogen, USA). Hi Script II Q RT Su-per Mix (Nanjing Novozan Biotechnology Co., Ltd.) was used to reverse transcribe the total RNA to obtain cDNA. Cham Q Universal SYBR q PCR Master Mix (Nanjing Novozan Biotechnology Co., Ltd.) was used for RT-qPCR. GAPDH is used as an endogenous reference. All primers were designed and synthesized by Shanghai Shenggong Biotechnology Co., Ltd. Related gene RT qPCR amplification primers can be found in Table S1.

### Statistical analysis

Statistical analysis was performed using GraphPad Prism 10 and R Studio 4.3.1. All data are expressed as mean ± SEM. The differences between two groups of independent samples were calculated using the unpaired Student’s t-test. The data analysis results were all corrected by FDR. The statistical significance level was set at *P* < 0.05; *<0.05, **<0.01, and ***<0.001.

## Results

### Identification and functional enrichment analysis of DEGs in depression and stroke

**Fig. 1:**
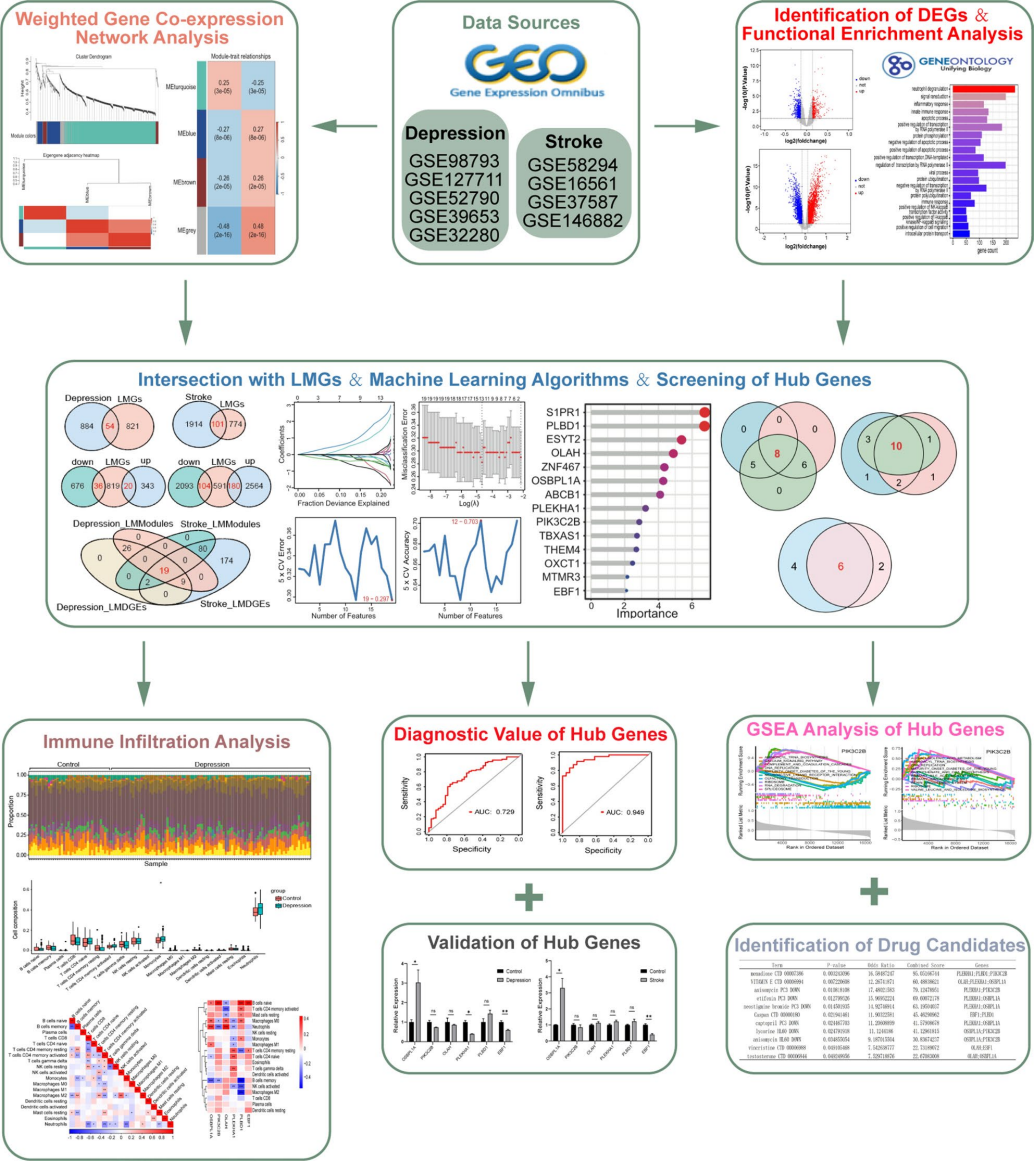
Flowchart of the study design

**Fig. 2:**
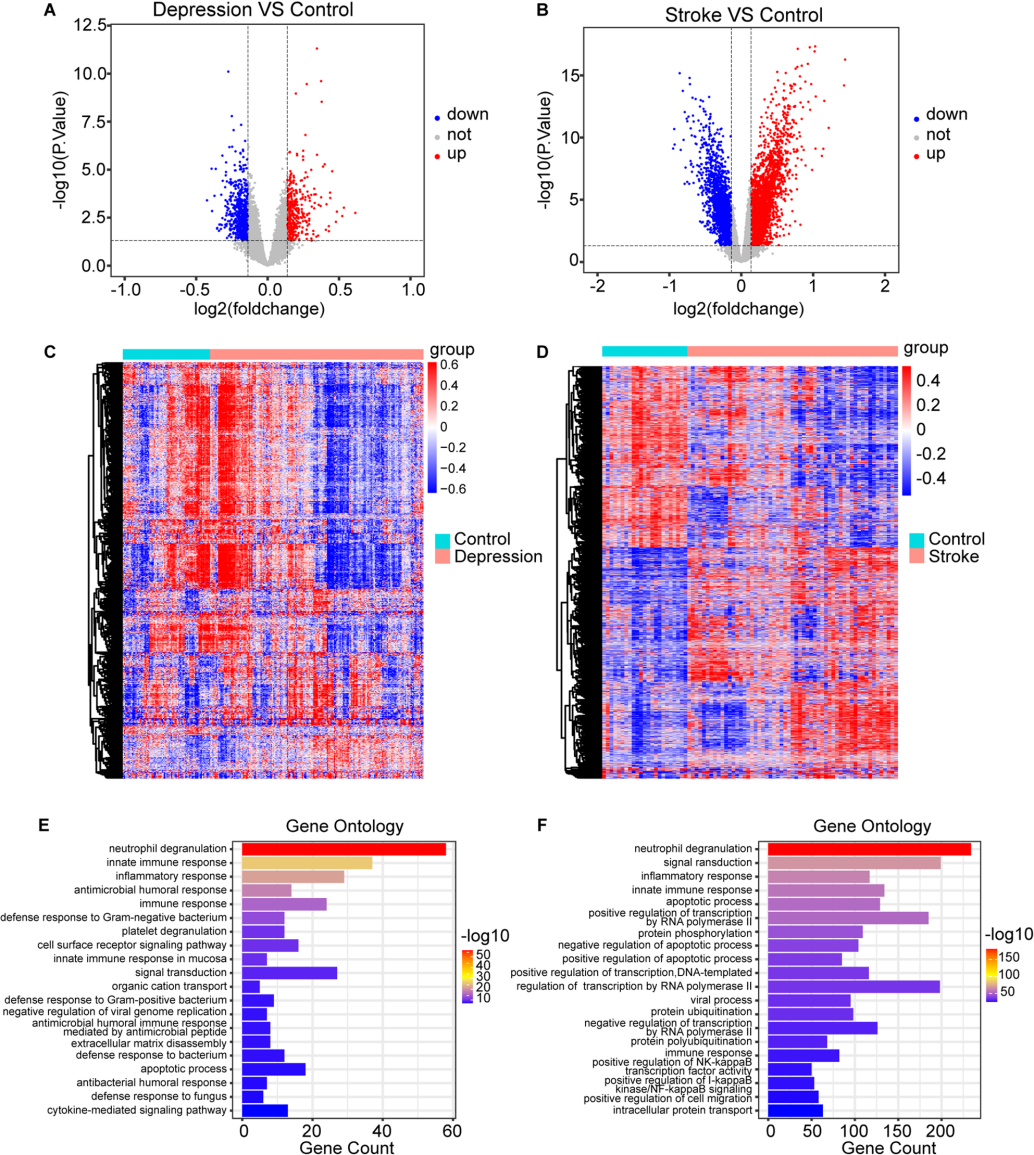
Identification of DEGs. Volcano plot of DEGs in depression group **A** and stroke group **B**. Heatmap of DEGs in depression group **C** and stroke group **D**. GO analysis of the up-regulated DEGs in depression group **E** and stroke group **F**

The analytic workflow of this study is shown in Fig. 1. The datasets GSE98793, GSE127711 and GSE52790 included a total of 186 patients with depression and 76 healthy controls for differential gene expression analysis. In this analysis, 1,075 DEGs were identified, of which 363 were upregulated and 712 were downregulated. The identified DEGs are illustrated in Fig. 2A and their expression patterns are visualized in the heatmap shown in Fig. 2C. Similarly, in the context of stroke we analyzed the datasets GSE58294, GSE16561, and GSE37587, which included 96 stroke patients and 47 healthy controls. This analysis revealed 4,941 DEGs, with 2,744 genes upregulated and 2,197 down-regulated in stroke. The volcano plot (Fig. 2B) and heatmap (Fig. 2D) depict the distribution and expression profiles of these DEGs associated with stroke.

GO and KEGG enrichment analyses were performed to characterize the potential functions and pathways associated with the identified DEGs in depression and stroke patients. In depression, GO enrichment analysis revealed that the upregulated DEGs were significantly enriched in terms related to “neutrophil degranulation” (Fig. 2E) while down-regulated DEGs were strongly associated with “regulation of transcription by RNA polymerase II” (Fig. S1A). The results of the GO enrichment analyses of stroke patients were similar to those of depression patients (Fig. 2, Fig. S1B). The KEGG analysis revealed that the DEGs of depression patients were involved primarily in signaling pathways such as “metabolic pathways”, “herpes simplex virus 1 infection”, and “complement and coagulation cascades” while the analysis of stroke highlighted the involvement of DEGs in pathways such as “metabolic pathways” (Fig. S1C–F).

### Weighted gene co-expression network analysis of depression and stroke

**Fig. 3:**
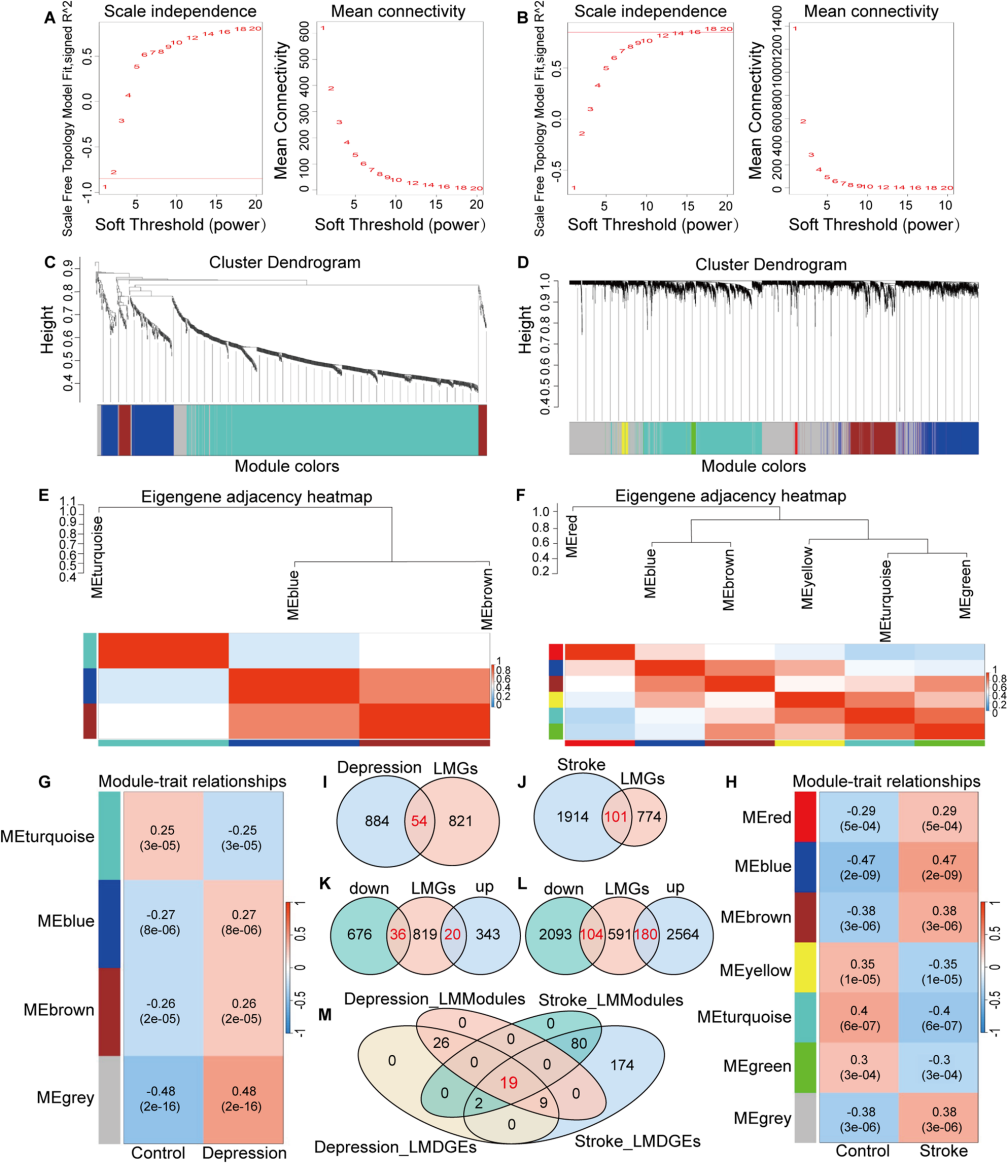
Weighted gene co-expression network analysis. Scaleless index and average connectivity of individual soft thresholds for depression **A** and stroke **B**. Gene co-expression modules with different colors under the gene tree in depression **C** and stroke **D** group. Dendrogram of ME and heatmap plot of the adjacencies of modules in depression group **E** and stroke group **F**, with red representing high adjacency whereas blue representing low adjacency. Correlation among modules and traits in depression **G** and stroke **H** group. Venn diagrams identify the intersections of key modules genes and LMGs of depression **I** and stroke **J**. Venn diagrams identify the intersections of DEGs and LMGs of depression **K** and stroke **L**. **M**. Overlapping genes of DEGs, LMGs and WGCNA module genes shown in the Venn diagram

WGCNA was conducted to search for key module genes and investigated the relationships between genes and the clinical characteristics of depression and stroke. Using soft thresholding, a coexpression network was constructed to identify key modules associated with each condition. We performed network topology analysis to ensure that the network adhered to the characteristics of a scale-free network (Fig. 3A). Through analysis of the original combined depression dataset, four gene modules were re-identified in total, including Turquoise (779 genes), Brown (55 genes), Blue (159 genes) and Gray (82 genes). The Gray module collected genes not assigned to any specific module and was excluded from further analyses. By calculating correlations between modules and clinical traits we found that the Blue module presented the strongest positive correlation with depression, while the Turquoise module presented the strongest negative correlation (Fig. 3C, E, G). As for depression patients, we performed network topology analysis for stroke patients (Fig. 3B). A total of 6 modules were identified after excluding the Gray module, ranging in size from 28 (Red) to 1,299 genes (Turquoise). With respect to the relationships between modules and clinical features, the Blue and Turquoise modules demonstrated the strongest correlations with stroke (Fig. 3D, F, H).

To understand the role of lipid metabolism in both diseases, 875 lipid metabolism genes were selected and intersected with significantly associated module genes in depression and stroke. This process yielded 54 genes related to depression and 101 genes related to stroke. By further intersecting DEGs with lipid metabolism genes, 56 genes were subsequently identified for depression, and 284 were identified for stroke. Finally, by combining these four intersections, 19 overlapping genes were identified, including ABCB1, AGPAT5, AKR1B1, ALOX5, BMX, EBF1, ELOVL4, ESYT2, MTMR3, OLAH, OSBPL1A, OXCT1, PIK3C2B, PLBD1, PLEKHA1, S1PR1, TBXAS1, THEM4, and ZNF467(as shown in Fig. 3I–M).

### Identification of comorbidity diagnostic genes using machine learning algorithms

**Fig. 4:**
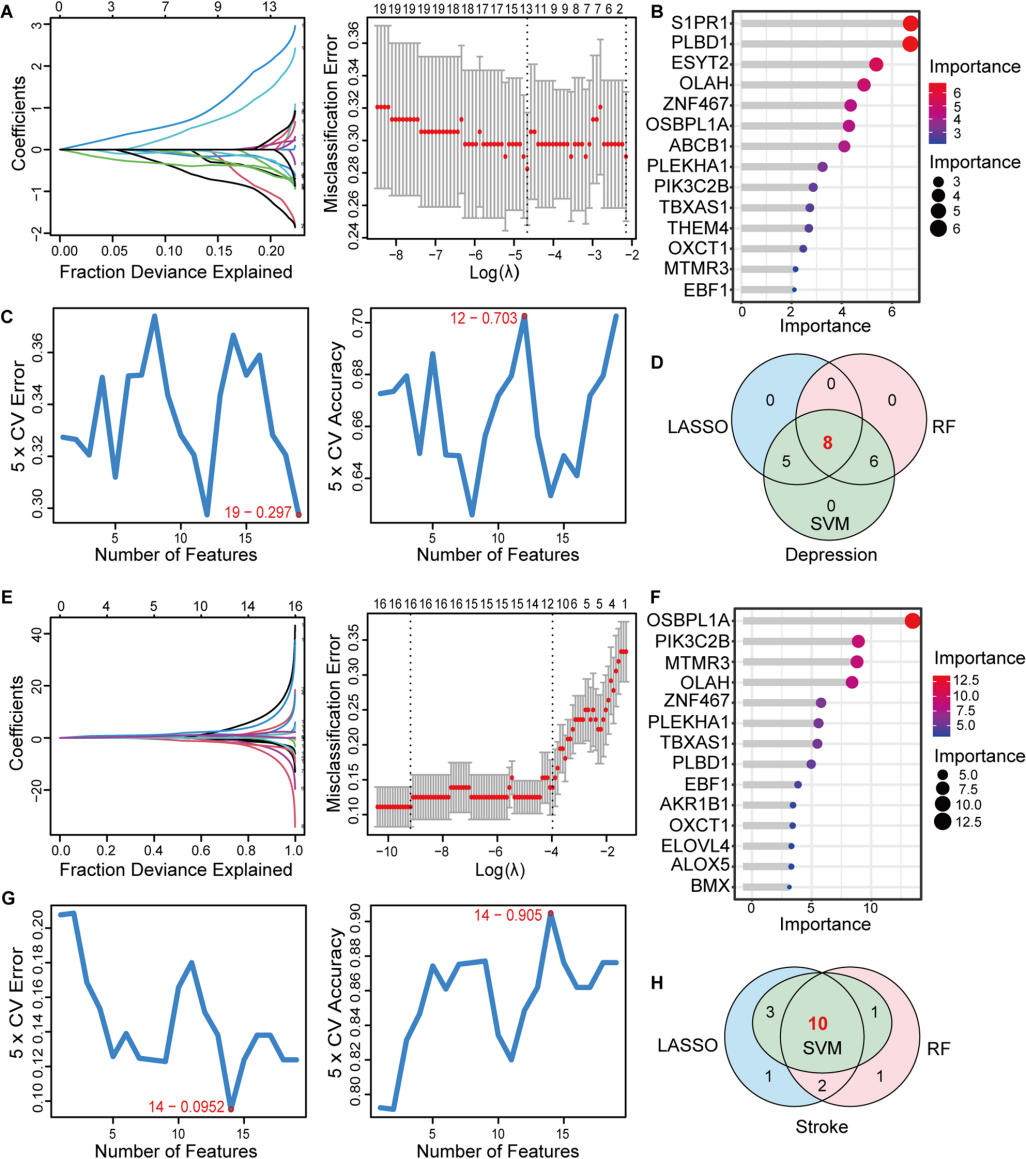
The screening of diagnostic genes with three machine learning algorithms. Coefficient profile plot of the LASSO model for depression **A** and stroke **E** showed the final parameter selection λ. The top 14 significant genes of depression **B** and stroke **F** according to the RF algorithm. Crosstalk genes were selected by using the SVM-RFE algorithm for depression **C** and stroke **G**. Venn diagram demonstrated eight candidate diagnostic genes in depression **D** and ten in stroke **H** by intersecting the results of three machine learning algorithms

To further pinpoint potential comorbidity diagnostic genes we employed SVM-RFE, RF, and LASSO machine learning algorithms. In the depression training group, LASSO analysis revealed 13 genes closely associated with the disease (Fig. 4A). The RF classifier filtered these genes, highlighting the top 14 genes based on importance (Fig. 4B). The SVM algorithm identified 19 genes with the lowest 5-point CV error and best 5-point CV accuracy (Fig. 4C). By intersecting the results of these three algorithms we identified 8 candidate diagnostic genes (Fig. 4D). Similarly, in the stroke training group we identified 14 genes using the SVM algorithm, 14 genes using the RF algorithm, and 16 genes using the LASSO algorithm. Consequently, we identified 10 overlapping genes specific to stroke (Fig. 4E–H).

### Diagnostic value and validation of hub diagnostic genes

**Fig. 5:**
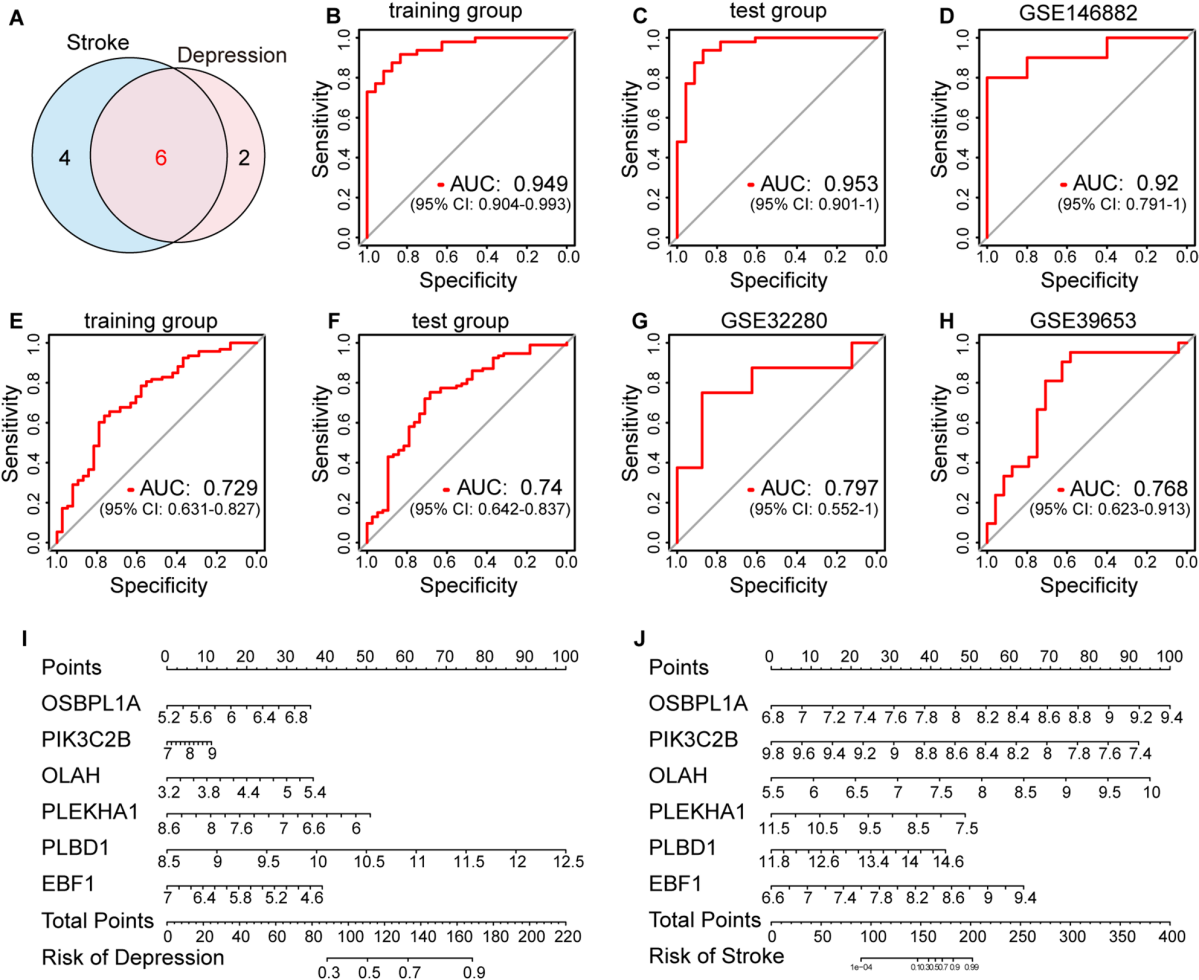
Validation of the shared diagnostic genes. **A**. The Venn plot showed the six shared diagnostic genes in depression and stroke. ROC curve of the diagnostic model constructed by six key genes in the training group **B** and the test group **C** of stroke. **D**. ROC curve of the diagnostic model in GSE146882. ROC curve of the diagnostic model in the training group **E** and the test group **F** of depression. ROC curve of the diagnostic model in GSE32280 **G** and GSE39653 **H**. The nomogram of depression **I** and stroke **J** was constructed based on the diagnostic biomarkers

Six genes (OLAH, OSBPL1A, PLBD1, EBF1, PIK3C2B, and PLEKHA1) were identified by intersecting hub genes from depression and stroke patients to construct a diagnostic model for further validation (Fig. 5A).

The differential expression of these shared diagnostic genes between the disease and healthy groups was analyzed. In the test sets of depression and stroke, OLAH, OSBPL1A, and PLBD1 presented increased expression in the disease groups, while EBF1, PIK3C2B, and PLEKHA1 presented decreased expression, consistent with the trends observed in the training group (Figure S2A–D). ROC analysis demonstrated the high specificity and sensitivity of the diagnostic model. The AUC values for stroke were 0.949 in the training set and 0.953 in the test set (Fig. 5B, C), and those for depression were 0.729 and 0.74 respectively (Fig. 5E, F). External validation using the stroke dataset GSE146882 resulted in an AUC of 0.92, indicating strong diagnostic capability (Fig. 5D). Similarly, in depression patients external independent validation sets GSE39653 (AUC = 0.768) and GSE32280 (AUC = 0.797) confirmed the model’s good diagnostic performance for distinguishing depression patients from healthy controls (Fig. 5G, H). The nomograms constructed with six key diagnostic genes for depression and stroke were diagnostically effective, as shown in Fig. 5I, J.

### Gene set enrichment analysis of diagnostic genes

**Fig. 6:**
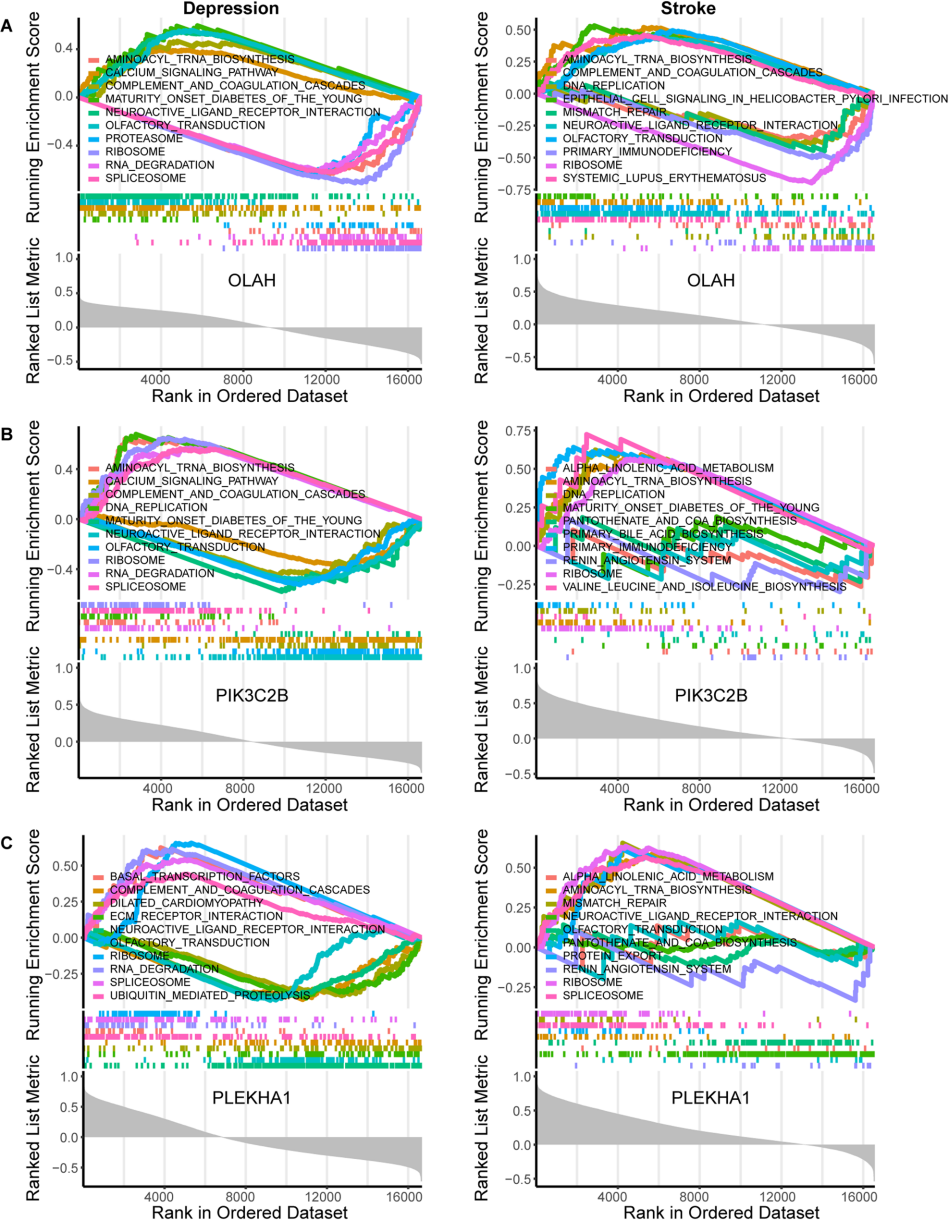
GSEA for the single diagnostic gene. **A**. GSEA analysis for OLAH in depression and stroke group. **B**. GSEA analysis for PIK3C2B in depression and stroke group. **C**. GSEA analysis for PLEKHA1 in depression and stroke group

To understand the potential pathways associated with each diagnostic gene in the comorbidity, GSEA was utilized. The top 5 positive and negative pathways were visualized using the “GSEA” package, revealing the following insights: OLAH affects multiple pathways in both diseases, for example “aminoacyl tRNA biosynthesis”, “ribosome”, “complement and coagulation cascades”, “neuroactive ligand receptor interaction”, and “olfactory transduction” (Fig. 6A). OSBPL1A influences “epithelial cell signaling in Helicobacter pylori infection” and the “nod-like receptor signaling pathway” (Fig. S3A). PLBD1 affected the “lysosome” pathway in both depression patients and stroke patients (Fig. S3B). Among the downregulated genes, EBF1 significantly influenced the “spliceosome” pathway in both depression and stroke patients (Fig. S3C). PIK3C2B affected pathways such as “maturity onset diabetes of the young”, “aminoacyl tRNA biosynthesis”, “DNA replication”, and “ribosome” (Fig. 6B). PLEKHA1 affected pathways such as “neuroactive ligand receptor interaction”, “olfactory transduction”, “ribosome”, and “spliceosome” (Fig. 6C). The “olfactory transduction” pathway can be influenced by all six genes in depression. Notably, most hub genes were associated with pathways related to “ribosome” and “aminoacyl tRNA biosynthesis” suggesting their potential importance in the comorbidity of depression and stroke.

### Immune infiltration analysis

**Fig. 7:**
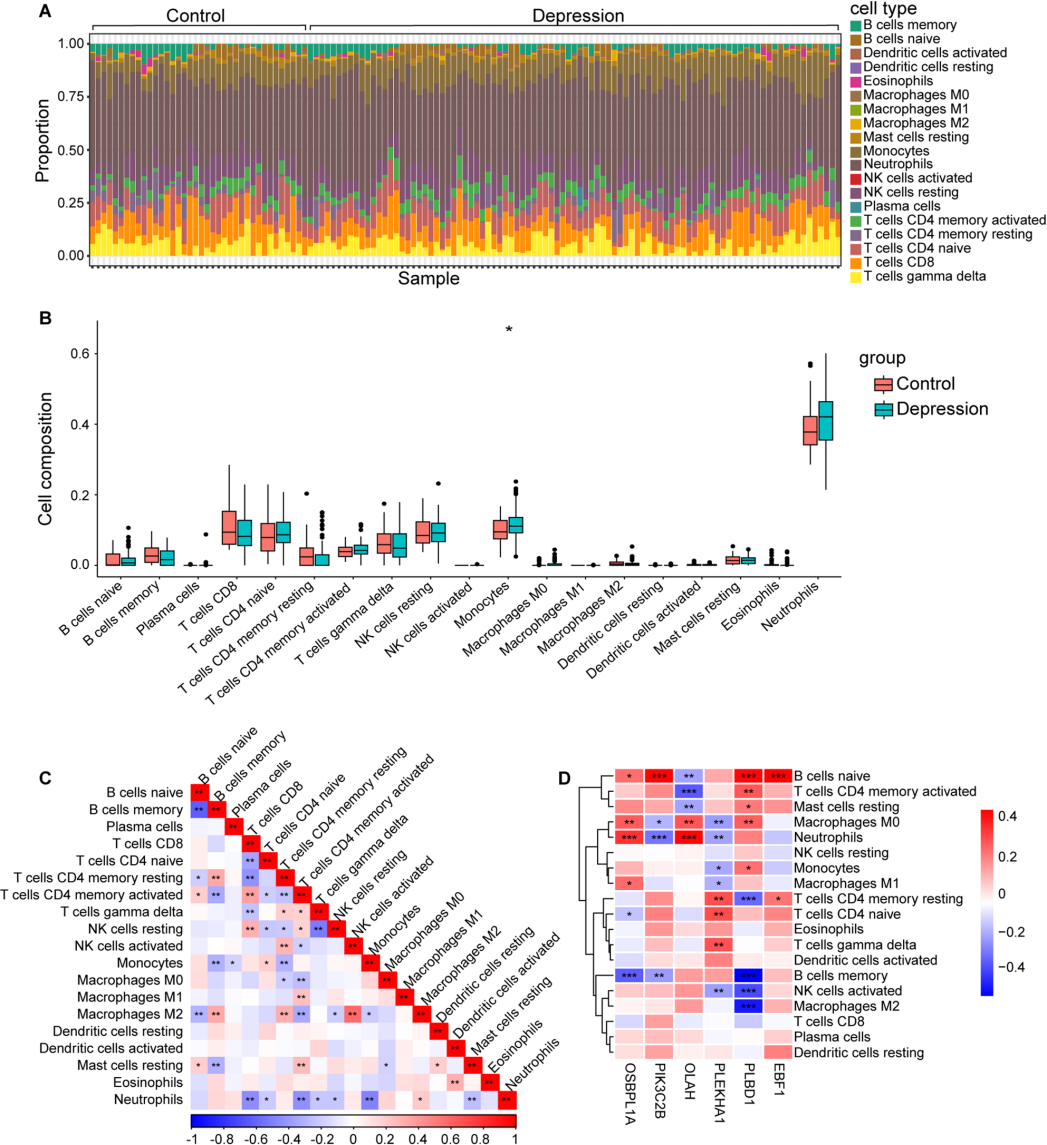
Immune infiltration analysis in the training group of depression. **A**. Immune cell infiltration map in each sample. **B**. Box plots show the comparison of immune cells between depression and control groups. **C**. The correlation of immune cells in depression revealed by the heatmap. **D**. Correlation between the six hub genes and infiltrating immune cells

**Fig. 8:**
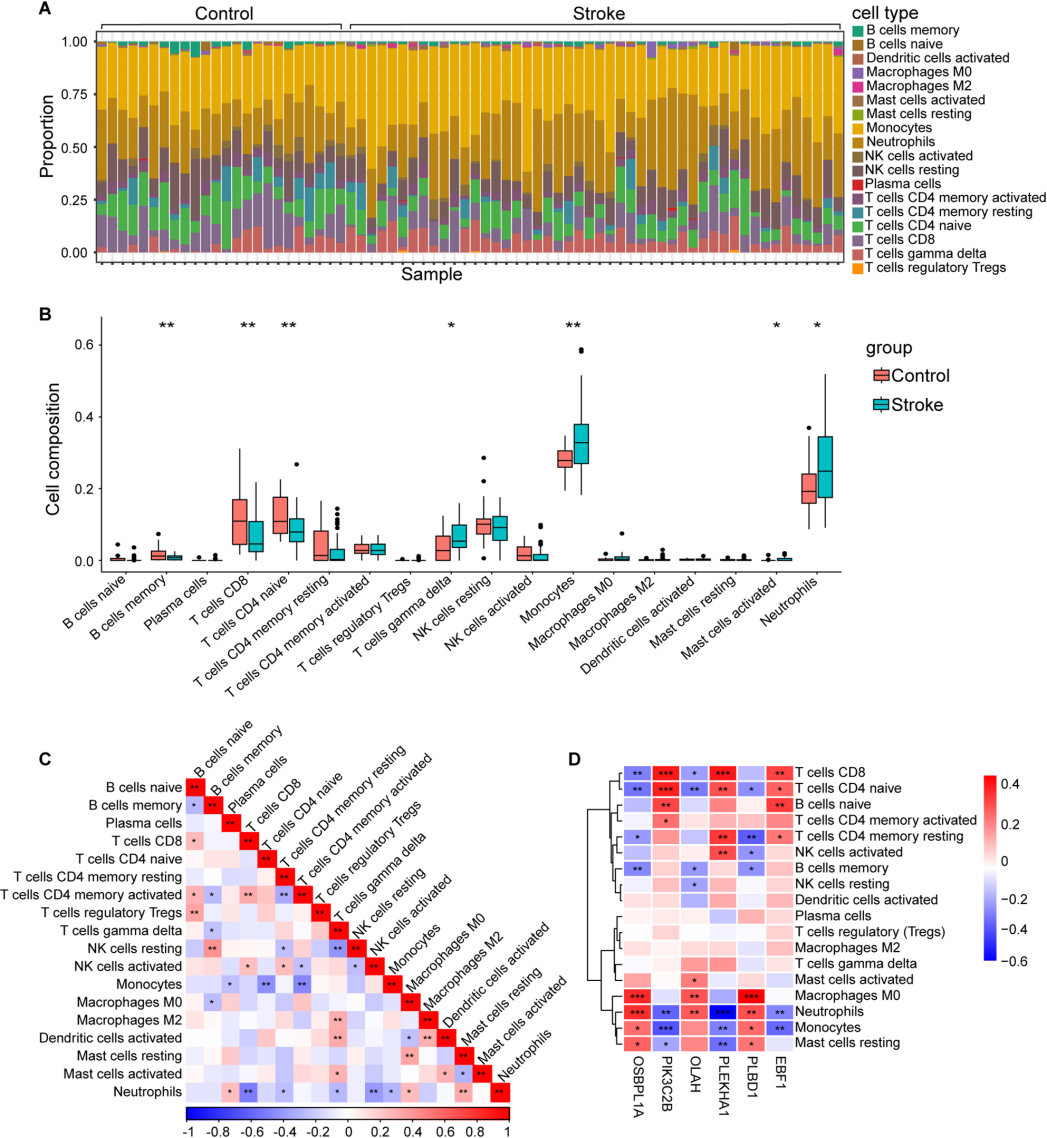
Immune infiltration analysis in the training group of stroke. **A**. Immune cell infiltration map in each sample. **B**. Box plots show the comparison of immune cells between stroke and control groups. **C**. The correlation of immune cells in stroke revealed by the heatmap. **D**. Correlation between the six hub genes and infiltrating immune cells

In investigations of the development of comorbidities, inflammation has emerged as a vital factor. Consequently, immune infiltration analysis was conducted on the training and test sets of depression and stroke patients using the CIBERSORT method, as shown in Figs. 7 and 8.

The proportions of each immune cell type in the samples were examined, yielding intriguing findings. Neutrophils were significantly present in both depression and stroke samples. Notably, monocytes were enriched in the combined stroke dataset. Observations of differences in immune cells between the disease samples and the respective control samples revealed that the numbers of neutrophils and monocytes were greater in the disease samples than in the normal samples while the number of T cells was lower. Correlations among immune cells were visualized by immune scores. A strong negative correlation between B-cell memory and naive B-cells in depression patients was observed. Similar negative correlation could be observed in stroke. Furthermore, a strong competitive effect between neutrophils and CD8 T cells in both diseases was detected. Further exploration focused on the associations of the 6 key diagnostic genes with immune infiltration revealed that most hub genes (OSBPL1A, OLAH, PIK3C2B, and PLEKHA1) exhibited significant correlations with neutrophil cells, with OSBPL1A displaying the most substantial positive correlation. Additionally, strong positive correlations were noted between EBF1 and naive B-cells, as well as between PLEKHA1 and resting memory CD4 T-cells. Similar results were observed in the test sets (Fig. S4, S5).

### Identification of drug candidates

To understand the potential impact of these genes on drug treatments, drug target enrichment analysis was conducted for the six shared diagnostic genes using the drug profile database. The findings are summarized in Table 2. Menadione and vitamin E were identified as potential targeting agents for three hub genes. Other drugs listed include anisomycin, etifenin, neostigmine, Caspan, captopril, lycorine, vincristine, and testosterone. These drugs can target two key diagnostic genes.

**Table 2:** Identification of drug candidates

Term	*P*-value	Odds Ratio	Combined score	Genes
menadione	0.003243096	16.58487247	95.05166744	PLEKHA1;PLBD1; PIK3C2B
vitamin E	0.007220608	12.26741871	60.48838621	OLAH; PLEKHA1; OSBPL1A
anisomycin	0.010818108	17.48021583	79.12478951	PLEKHA1;PIK3C2B
etifenin	0.012799526	15.96952224	69.60072178	PLEKHA1;OSBPL1A
neostigmine bromide	0.014501935	14.92746914	63.19504037	PLEKHA1;OSBPL1A
caspan	0.021941461	11.90322581	45.46290962	EBF1;PLBD1
captopril	0.024467703	11.20608899	41.57908678	PLEKHA1;OSBPL1A
lycorine	0.024791918	11.1244186	41.12961815	OSBPL1A; PIK3C2B
anisomycin	0.034855054	9.187015504	30.83674237	OSBPL1A; PIK3C2B
vincristine	0.049105468	7.542638777	22.73189072	OLAH; EBF1
testosterone	0.049249956	7.529718876	22.67083008	OLAH; OSBPL1A

### Validation of hub genes

**Fig. 9:**
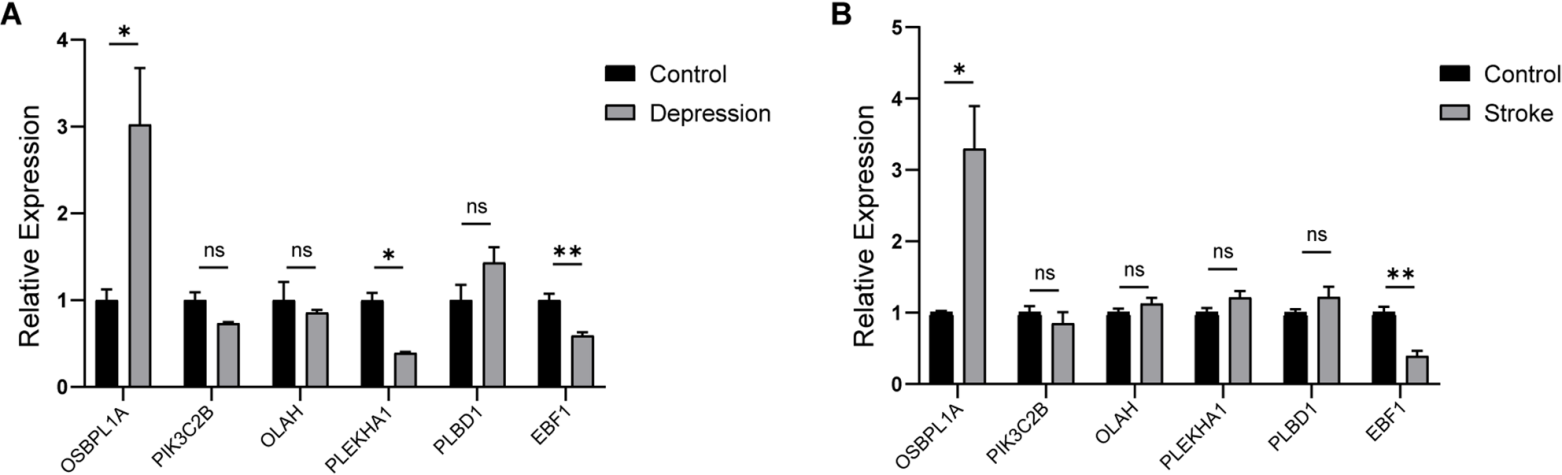
Validation of hub genes by RT-qPCR. **A**. Expression levels of six hub genes in serum samples of normal control (*n* = 3) and depression patients(*n* = 3). **B**. Expression levels of six hub genes in serum samples of normal control (*n* = 3) and stroke patients (*n* = 3)

RT-qPCR was conducted on blood samples from patients with stroke, depression and healthy individuals to verify the gene expression levels of six diagnostic biomarkers. The experimental results show that, except for OLAH, the expression patterns of these hub genes in depression are consistent with the aforementioned analysis results (Fig. 9A). The expression of OSBPL1A and PLBD1 is upregulated, while the expression of EBF1, PIK3C2B and PLEKHA1 is downregulated. Among stroke patients, except for PLEKHA1, the expression patterns of the other genes were also consistent with the analysis results (Fig. 9B). In addition, both OSBPL1A and EBF1 showed significant differences in stroke and depression.

## Discussion

The connection between mood disorders such as depression and bipolar disorder (BD) and metabolic dysfunction, termed “metabolic-mood syndrome”, underscores the heightened risk of depression in individuals with obesity and related metabolic disorders [26, 27]. Abnormal blood glucose levels further increase suicide risk in those with depression. Studies suggest that Bifidobacterium breve CCFM1025 can alleviate depression by regulating tryptophan metabolism, while oxidative stress is a key factor in neurodegeneration in depression [28, 29]. Similarly, metabolic issues not only increase the risk of stroke but also impact its development and prognosis, influencing processes such as post-stroke glial scar formation [30]. Glucose metabolism abnormalities are common in acute stroke patients, emphasizing the importance of interventions such as diet-induced weight loss to enhance recovery [31–33]. Abnormalities in the GLU/GABA to GLN metabolic cycle are linked to post-stroke depression pathogenesis, suggesting potential therapeutic targets [34].

Lipid metabolism is crucial for understanding and treating both depression and stroke. Erythrocyte membrane fatty acid levels show promise as biological markers correlated with psychiatric symptom severity [6, 35]. In stroke, long-chain acylcarnitines (LCACs) induce astrocytic mitochondrial dysfunction, impacting neuronal damage and serving as potential acute ischemic stroke biomarkers [36]. rtPA treatment influences brain-wide lipid metabolism changes, affecting stroke prognosis [37]. Plasma and fecal metabolomics underscore the importance of lipid metabolism, especially DHA, in predicting PSD [38].

Integrative bioinformatics analysis revealed six shared lipid metabolism-related genes (OLAH, OSBPL1A, PLBD1, EBF1, PIK3C2B, and PLEKHA1) associated with both stroke and depression. ROC analysis revealed the value of these genes in the diagnosis of these two diseases. The identified diagnostic genes, such as OSBPL1A, EBF1, PIK3C2B, and PLBD1, have intriguing associations with stroke. For example, the involvement of OSBPL1A in cholesterol transport and the development of atherosclerotic lesions raises the possibility of its impact on stroke risk [39, 40]. EBF1, known for its pivotal role in adipogenesis and immune system development, has also been linked to various metabolic and inflammatory pathways, emphasizing its importance in cardiovascular diseases [41–45]. Similarly, the connection of PIK3C2B to the PI3K/Akt pathway highlights its potential for post-stroke neuroprotective effects [46]. Similarly, PLBD1 has emerged as a notable marker for aneurysmal subarachnoid hemorrhage, highlighting its relevance in severe forms of stroke [47, 48]. However, the nuanced relationships between these genes and their roles in depression or stroke warrant further exploration to elucidate their comprehensive implications.

GSEA revealed intriguing insights into these diagnostic genes. The enrichment of the “olfactory transduction” pathway among genes associated with depression suggests a potential link between olfaction and depression. Olfaction’s direct connections to brain regions regulating cognition and emotions underscore its significance in mental health [49–51]. Furthermore, the shared risks between olfactory loss and conditions such as diabetes, hypertension, cardiovascular ailments, and obesity—factors influencing stroke—highlight common underlying pathways that merit further investigation [52–55]. Moreover, the “aminoacyl tRNA biosynthesis” pathway enrichment observed in the stroke group points toward significant mechanisms underlying stroke pathogenesis. Similar enrichments found in PSD patients in prior research further emphasize the relevance of these pathways in stroke-related complications [56]. By delving deeper into the associations of these diagnostic genes with depression and stroke, future research can reveal critical insights into their pathogenic roles and pave the way for targeted therapeutic interventions in these complex disorders.

Immune infiltration analyses emphasized the intertwined roles of neutrophils and monocytes in the copathogenesis of these conditions, offering insights for early diagnosis and tailored treatment strategies for this comorbidity. Neutrophils are swiftly recruited to the injured brain after stroke and their increased presence is linked to poor recovery and disrupted vascular repair. Targeting neutrophils to reduce harmful neutrophil extracellular trap formation could be beneficial for stroke treatment [57–62]. In depression, elevated neutrophil levels and decreased lymphocyte counts characterize immune cell alterations [63]. The neutrophil-to-lymphocyte ratio serves as a biomarker for PSD and there is a correlation between depression severity and this ratio [64, 65]. Selective serotonin reuptake inhibitors can reduce neutrophil percentages and monocyte counts in depressed patients, indicating that neutrophils are potential therapeutic targets for depression and stroke comorbidity [66]. Changes in monocytes are associated with stroke occurrence and development. Monocytes contribute to inflammatory responses, worsen brain damage, and affect blood‒brain barrier integrity. They play a role in predicting stroke prognosis, and their function can shift from enhancing the immune response to reducing inflammation during ischemic stroke [67–70]. Cell therapies targeting monocytes show promise in promoting recovery after cerebral ischemia [71]. Our GSEA and immune infiltration analyses shed light on shared pathways and the involvement of immune cells in both depression and stroke, emphasizing the crucial roles of olfactory transduction, neutrophils, and monocytes.

By exploring lipid metabolism-related genes as potential drug targets, we discovered an association between testosterone deficiency and depressive symptoms, and corresponding complementary therapies have promising results for specific depressive conditions [72–75]. Additionally, drugs such as losartan and captopril have the potential to improve post-stroke outcomes in stroke-prone hypertensive rats, suggesting therapeutic benefits for depression and stroke [76, 77].

Despite our insights, this study has several limitations such as the lack of comprehensive comorbidity data for depression and stroke, hindering a deeper exploration of their associations. The reliability of immune cell analysis under disease conditions and the complex molecular mechanisms by which central genes cause disease remain unclear, necessitating further experimental and clinical research to validate their functions and the efficacy of potential therapeutic drugs. Furthermore, this study is limited by the absence of some clinical data in public databases and is unable to fully control the possible confounding effects. In the future, we will further verify and enhance the reliability of this research result by collecting more complete datasets.

In conclusion, our study validates six signature genes related to lipid metabolism in depression and stroke, highlighting their link with immunity, particularly neutrophils. Understanding this relationship provides insights into common molecular mechanisms in depression and stroke, potentially unveiling new targets for diagnosing and treating this comorbidity from a metabolic perspective.

## Supplementary Information

Below is the link to the electronic supplementary material.

Supplementary Material 1

## Data Availability

The original contributions analyzed in the study are publicly available. Datasets used can be downloaded from the GEO database (http://www.ncbi.nlm.nih.gov/geo/). The depression group includes five datasets with GSE98793, GSE127711, GSE52790, GSE39653, and GSE32280. GSE58294, GSE16561, GSE37587, and GSE146882 are involved in the stroke group. Further inquiries can be directed to the corresponding authors.

## Abbreviations

MDD: Major depressive disorder
NCBI: National center for biotechnology information
GEO: Gene expression omnibus database
WGCNA: Weighted gene coexpression network analysis
ROC: Receiver operating characteristic
DEGs: Differentially expressed genes
GSEA: Gene set enrichment analysis
PSD: Post-stroke depression
BBB: Blood‒brain barrier
LMGs: Lipid metabolism related genes
GO: Gene ontology
KEGG: Kyoto encyclopedia of genes and genomes
TOM: Topological overlap matrix
MSigDB: Molecular signature database
DSigDB: Drug signature database
LASSO: Least absolute shrinkage and selection operator
SVM-RFE: Support vector machine-recursive feature elimination
RF: Random forest
AUC: Area under the curve
NES: Normalized enrichment score
BD: Bipolar disorder
LCACs: Long-chain acylcarnitines
