# Supplementary material for: Insights into the interplay between stroke and depression through lipid metabolism-related diagnostic genes
Source: Mol Brain. 2026 Jan 18;19:17. doi: 10.1186/s13041-026-01275-5 (PMC12930612; doi:10.1186/s13041-026-01275-5)
Supplement: Supplementary file 1 — Supplementary Material 1 [file 13041_2026_1275_MOESM1_ESM.docx]

**Table S1** Primer sequences

| **Gene** | **Forward primer** | **Reverse primer** |
| --- | --- | --- |
| OSBPL1A | TTGCTGTATCTGCTGTTGCTTCTC | AATCCAAGGTCATCTCGCACTAATTC |
| PIK3C2B | GCTTGCCTGCCTGACATCTATG | ACAGCCATACGACGCACCTC |
| OLAH | GAGAGGAGACCAACCTAAGAGAACC | CCATCCAGGGAAAGCAAATCAGC |
| PLEKHA1 | ATCAGCAGGACCTAGTGGAATGG | ATGGCGACTTAGGTTATCAGAATTAGG |
| PLBD1 | GTGACCAAGGGAAAGTGACTGATAC | CACGGCAGCAGATGGTATTACAG |
| EBF1 | GATAAGGACGGAGCAGGATTTCTAC | GACTCGGCACATTTCTGGGTTC |
| GAPDH | GTGGACCTGACCTGCCGTCTAG | GAGTGGGTGTCGCTGTTGAAGTC |


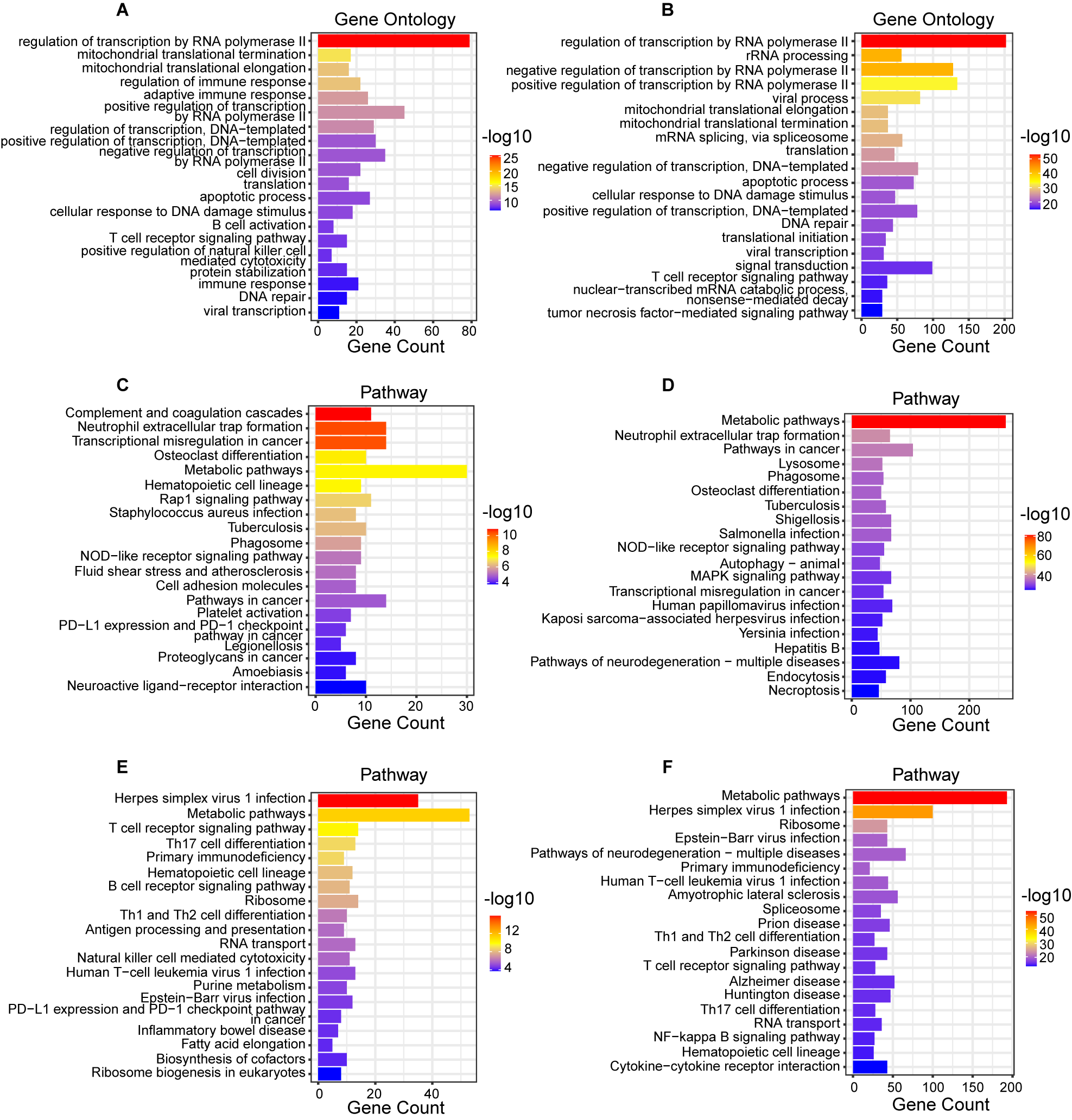


**Figure S1** Functional enrichment analysis. GO analysis of the down-regulated DEGs in depression group (A) and stroke group (B). KEGG analysis of the up-regulated DEGs in depression group (C) and stroke group (D). KEGG analysis of the down-regulated DEGs in depression group (E) and stroke group (F).


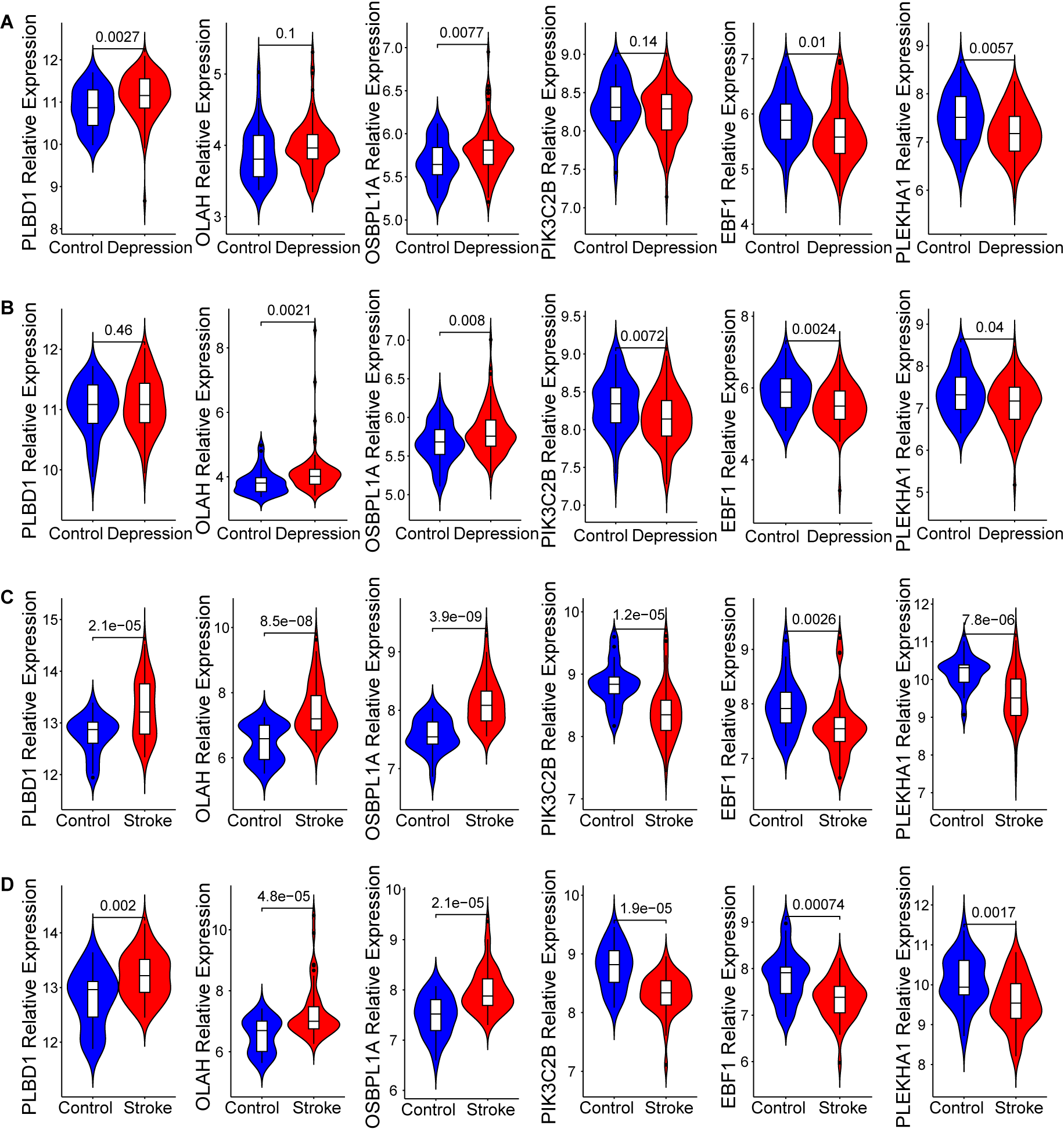


**Figure S2** Differential expressions of shared diagnostic genes. Expression levels of six hub genes in the training group and test group of depression (A, B) and stroke (C, D).


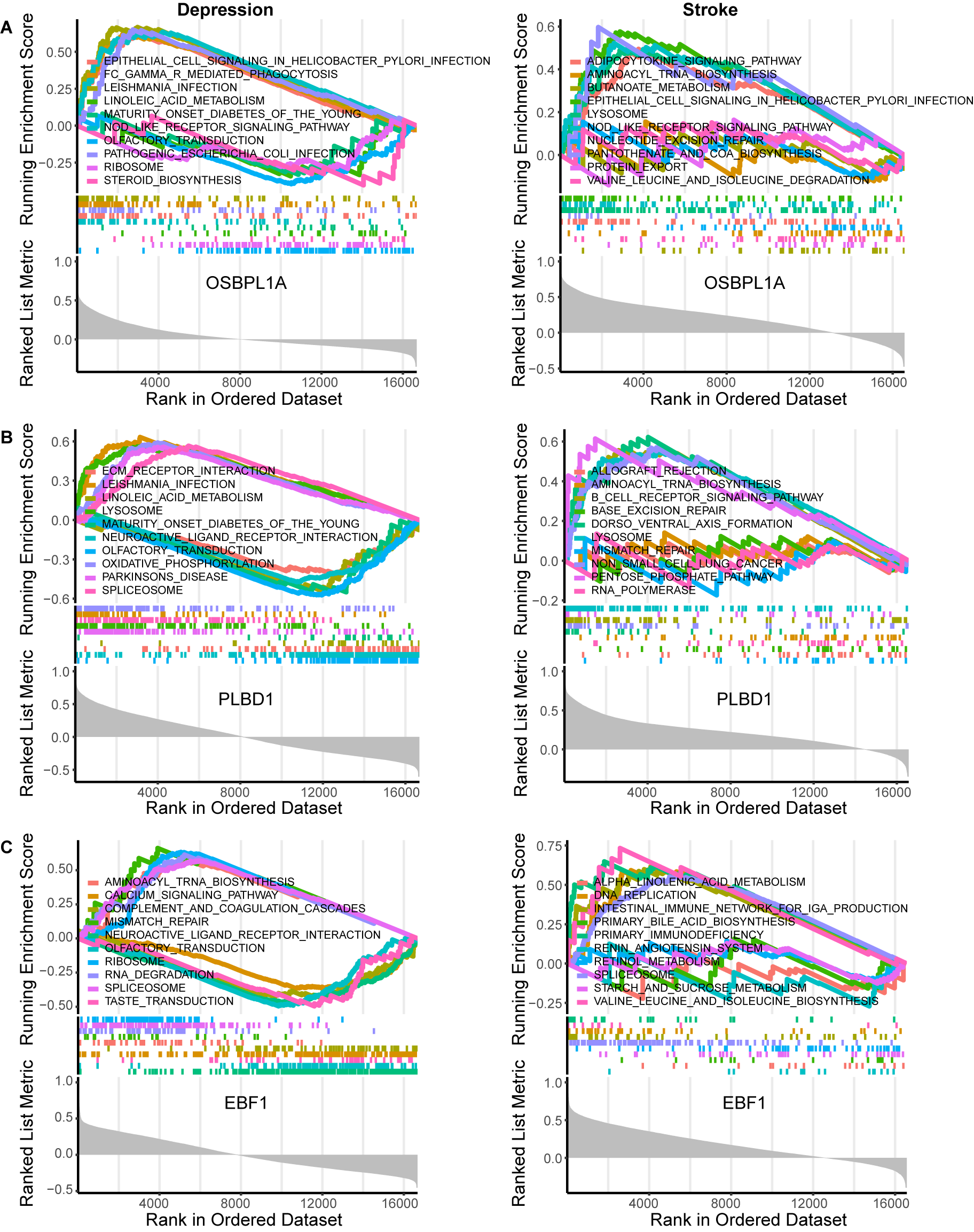


**Figure S3** GSEA for the single diagnostic gene. (A). GSEA analysis for OSBPL1A in depression and stroke group. (B). GSEA analysis for PLBD1 in depression and stroke group. (C). GSEA analysis for EBF1 in depression and stroke group.


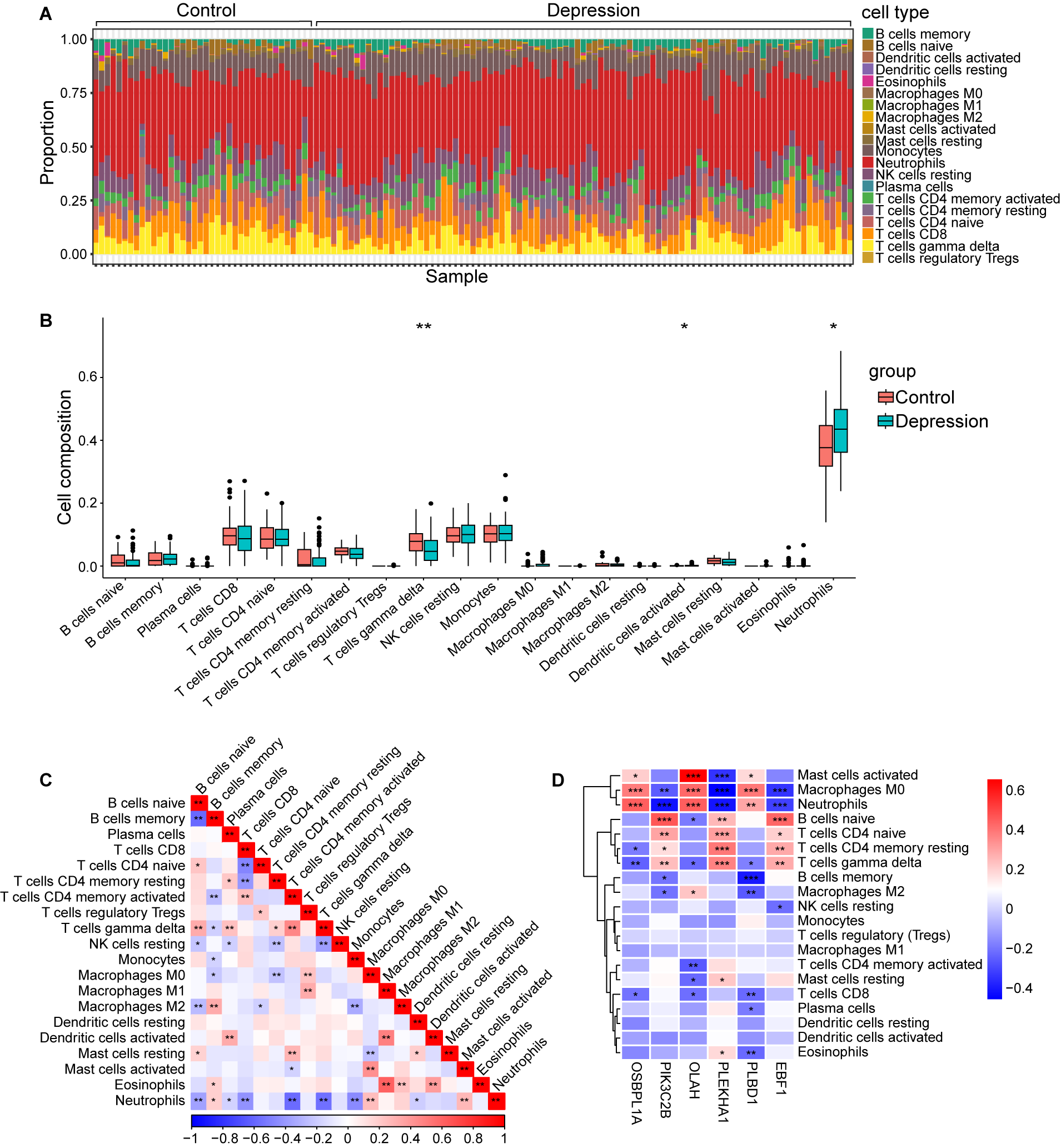


**Figure S4** Immune infiltration analysis in the test group of depression. (A). Immune cell infiltration map in each sample. (B). Box plots show the comparison of immune cells between depression and control groups. (C). The correlation of immune cells in depression revealed by the heatmap. (D). Correlation between the six hub genes and infiltrating immune cells.


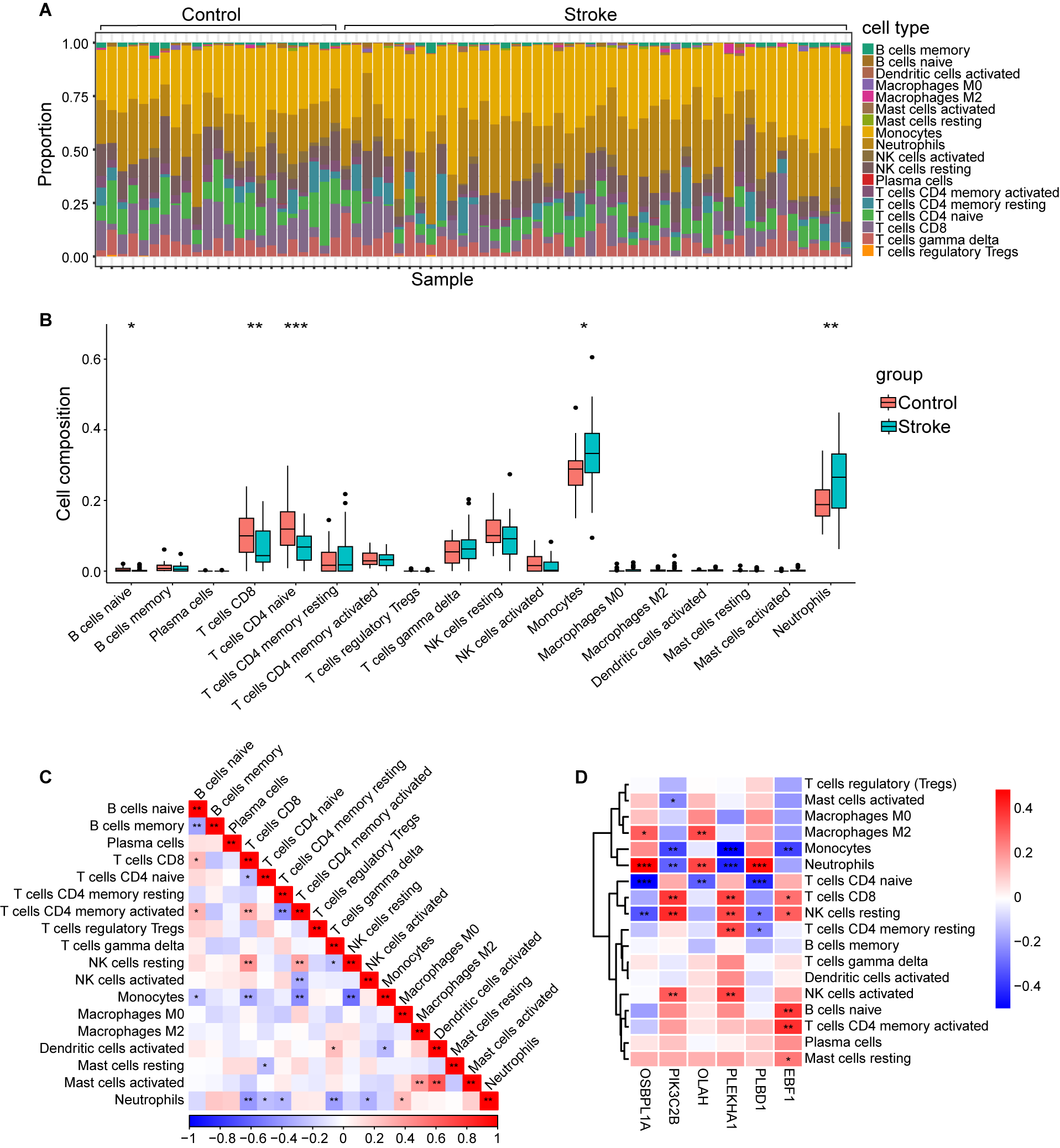


**Figure S5** Immune infiltration analysis in the test group of stroke. (A). Immune cell infiltration map in each sample. (B). Box plots show the comparison of immune cells between stroke and control groups. (C). The correlation of immune cells in stroke revealed by the heatmap. (D). Correlation between the six hub genes and infiltrating immune cells.
